# P-1680. Retrospective Review of Adherence to Institutional Pneumonia Antimicrobial Guidelines at Hospital Admission

**DOI:** 10.1093/ofid/ofae631.1846

**Published:** 2025-01-29

**Authors:** Bailey Burnette, Jesse M Thompson, Becky Reece, Jordan R Burnette, Catessa A Howard

**Affiliations:** WVU Medicine, Morgantown, West Virginia; West Virginia University, Morgantown, West Virginia; West Virginia University, Morgantown, West Virginia; U.S. Department of Veterans Affairs, Morgantown, West Virginia; West Virginia University Medicine, Morgantown, West Virginia

## Abstract

**Background:**

Literature supports the importance for institutions to have antimicrobial guidance accessible for providers in order to improve patient outcomes and antimicrobial stewardship (AMS). However, guideline adherence when prescribing for community acquired infections tends to be poor and includes more broad-spectrum antibiotics than required. We performed a review to assess adherence to our institutional pneumonia guidelines.

**Figure:**
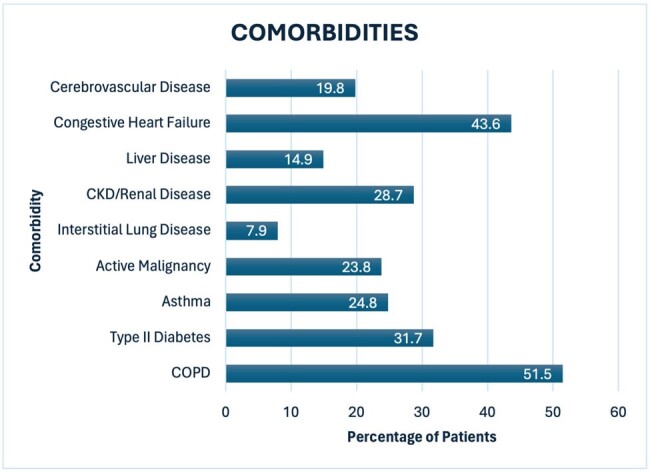

**Methods:**

A retrospective chart review was conducted on all adult patients admitted to a Medicine or Hospitalist team at WVU Hospitals with an admission diagnosis of community acquired pneumonia (CAP)/pneumonia between January – June 2023. Demographic and clinical characteristics were collected as well as outcomes of interest: empiric antimicrobials and adherence to our institutional pneumonia guidelines. Data was collected and analyzed via WVU REDCap.

**Figure:**
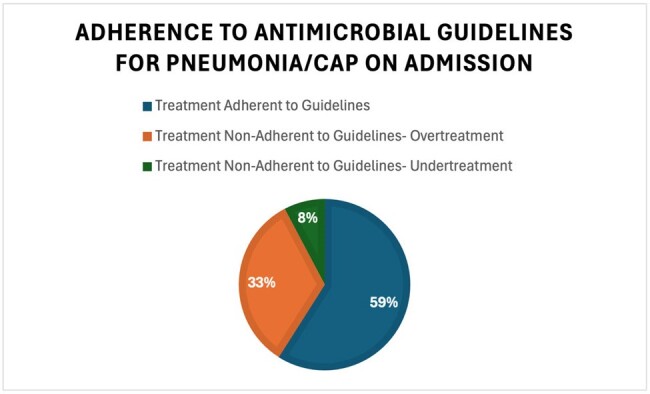

**Results:**

We identified 154 patients with admission diagnosis of CAP to internal medicine services; however, 37 were excluded, as they were transfers from the ICU or outside facilities. Demographics showed 49.4% of the patients were female and median age was 69 years. 30% received antibiotics in preceding 90 days, with 77% of these for prior pneumonia. Identified comorbidities included: COPD (51.5%), Type II Diabetes (31.7%), CHF (43.6%), active malignancy (23.8%) and immunosuppressed (35.6%) (Figure 1). The median Patient Severity Index score was 91 (26-277). Adherence to institutional guidelines was 59% and of those non-adherent, 81% were over treated (Figure 2). Frequency of appropriate antibiotic use included: 24.8% ampicillin/sulbactam, 52.1% doxycycline, 33.3% ceftriaxone, and 12.8% azithromycin. Most common inappropriate antibiotic use included cefepime 28.2% and vancomycin 23.9%.

**Conclusion:**

At 59% adherence to our pneumonia guidelines, this data shows there is a need for education and increased accessibility to institutional guidelines. Our QI intervention will include educational sessions on AMS guidelines and our CAP order panel, how to access the guidelines (via AMS app or webpage) and placement of guideline links within antibiotic orders. This study will also serve as a basis for assessing empiric treatment of other common infections and adherence to institutional AMS guidelines.

**Disclosures:**

**All Authors**: No reported disclosures

